# UAV Photogrammetry under Poor Lighting Conditions—Accuracy Considerations

**DOI:** 10.3390/s21103531

**Published:** 2021-05-19

**Authors:** Pawel Burdziakowski, Katarzyna Bobkowska

**Affiliations:** Department of Geodesy, Faculty of Civil and Environmental Engineering, Gdansk University of Technology, Narutowicza 11-12, 80-233 Gdansk, Poland; katarzyna.bobkowska@pg.edu.pl

**Keywords:** UAV, photogrammetry, night, model, light pollution

## Abstract

The use of low-level photogrammetry is very broad, and studies in this field are conducted in many aspects. Most research and applications are based on image data acquired during the day, which seems natural and obvious. However, the authors of this paper draw attention to the potential and possible use of UAV photogrammetry during the darker time of the day. The potential of night-time images has not been yet widely recognized, since correct scenery lighting or lack of scenery light sources is an obvious issue. The authors have developed typical day- and night-time photogrammetric models. They have also presented an extensive analysis of the geometry, indicated which process element had the greatest impact on degrading night-time photogrammetric product, as well as which measurable factor directly correlated with image accuracy. The reduction in geometry during night-time tests was greatly impacted by the non-uniform distribution of GCPs within the study area. The calibration of non-metric cameras is sensitive to poor lighting conditions, which leads to the generation of a higher determination error for each intrinsic orientation and distortion parameter. As evidenced, uniformly illuminated photos can be used to construct a model with lower reprojection error, and each tie point exhibits greater precision. Furthermore, they have evaluated whether commercial photogrammetric software enabled reaching acceptable image quality and whether the digital camera type impacted interpretative quality. The research paper is concluded with an extended discussion, conclusions, and recommendation on night-time studies.

## 1. Introduction

Low-level air photogrammetry using unmanned aerial vehicles (UAVs) has attracted huge interest from numerous fields over the last ten years. Photogrammetric products are used in various economic sectors, and thus intensively contribute to their growth. This situation primarily results from the development and widespread availability of UAVs equipped with good-quality non-metric cameras, the development of software base and easy-to-use photogrammetric tools, as well as increased computing power of personal computers. Despite the already widespread use of the aforementioned techniques, there is still a large number of issues associated with the processing of low-level photogrammetry products. This is mainly influenced by a relatively young age of this technology, the dynamic development of sensor design technology and modern computing methods. It can be stated without doubt that the complete potential of photogrammetry has not yet been fully discovered and unleashed, which is why scientists and engineers are constantly working on improving and developing the broadly understood UAV photogrammetry.

Works in the field of developing UAV measurement technologies are conducted concurrently on many levels. Scientists quiet rightly focus on selected elements of the entire photogrammetric product process, studying particular relationships, while suggesting new and more effective solutions. Research in the field of UAV photogrammetry can be divided into several mainstreams, with the main ones including:

- Carrier system technology and techniques [1,2,3,4]: Works in this group focus on improving the navigation-wise aspects of flight execution, georeference accuracy, or sensor quality in order to achieve even better in-flight performance, flight time and stability [5,6,7], as well as the accuracy of navigation systems feeding their data to measuring modules [8,9].
- Optimization of photogrammetric product processes [10,11,12,13]: Scientists look at processes ongoing at each stage of image processing and suggest optimal recommendations in terms of acquisition settings, image recording format [14], flight planning, or application of specific software settings [15].
- Evaluating the quality of results obtained using UAV photogrammetry [16,17]: These analyses address the issues of errors obtained for photogrammetric images and products, based on applied measuring technologies (from the acquisition moment, through data processing using specialized software).
- Focusing on the development of new tools improving the quality of low-level images [18,19,20]: Studies in this group involve a thorough analysis of the procedure of acquiring and processing UAV images and suggest new, mainly numerical, methods for eliminating identified issues [21,22,23]. As it turns out, photos taken from a dynamically moving UAV under various weather and UAV lighting conditions exhibit a number of flaws. These flaws result directly from the acquisition method and impact photogrammetric product quality.
- Showing new applications and possibilities for extracting information from spatial orthophotoimages based on photos taken in the visible light range [24,25,26,27] and by multi-spectral cameras [28,29,30]: Unmanned aerial vehicles are able to reach places inaccessible to traditional measurement techniques [31,32]. However, they carry an incomplete spectrum of sensors onboard, due to their restricted maximum take-off mass (MTOM). Therefore, scientists focus on methods that enable extracting significant information from this limited number of sensors and data, e.g., only from a single camera, but used at various time intervals [33] or a limited number of spectral channels in cameras used on the UAV.
- Presenting new photogrammetric software and tools [34,35,36]: The increasing demand for easy-to-use software obviously results in the supply of new products, both typically commercial and non-commercial products. New technologies and methods are developed in parallel.
- Using sensory data fusion [37,38,39]: The issue in this group is the appropriate harmonization of data obtained from a dynamically moving UAV and other stationary sensors. Very often, these data have a slightly different structure, density, or accuracy, e.g., integration of point cloud data obtained during a photogrammetric flight, terrestrial laser scanning, and bathymetric data [40,41].

The above research, focusing strictly on specified certain narrow aspects of developing a photogrammetric product based on data acquired from an unmanned aerial vehicle, translate into further application studies, case studies, and new practical applications. Naturally, the most populated group of application studies are works addressing the issue of analysing the natural environment and urban areas. Popular application-related research subjects include analysing the wood stand in forestry [42,43,44,45], supporting precise agriculture and analysing the crop stand [46,47,48], and geoengineering analyses for the purposes of landform change and landslide analyses [49,50,51,52].

As evidenced above, the application of low-level photogrammetry is very broad, and studies in this field are conducted in many aspects. It should be noted that all the aforementioned research is based on image data acquired during the day, which seems natural and obvious. However, the authors of this paper draw attention to the potential and possible use of UAV photogrammetry during the darker time of the day. Previously, the potential of night-time images has not been yet widely recognized, since correct scenery lighting or a lack of scenery light sources remain obvious issues [53].

Studies dealing with night-time photogrammetry that point to the potential of such photos are still a niche topic [54]. A good example is the case of images inside religious buildings, obtained during the night and supported by artificial lighting. Such methods are used in order to improve the geometric model quality of the studied buildings through avoiding reflections and colour changes caused by sunlight penetrating into the interior through colourful stained-glass windows [55]. Similar conclusions were drawn by the authors of [56], who studied the issues associated of modelling building facades. The dark time of the day favours background elimination and extracting light sources. This property was utilized by the authors of [57], who used light markers built from LEDs to monitor the dynamic behaviour of wind turbines. This enabled to achieve a high contrast of reference light points on the night-time photos, which improved their location and identification. Similar, too, was the case with analysing landslide dynamics [58]. Santise et al. [59] focused on analysing the reliability of stereopairs taken at night, with various exposure parameters, for the purposes of geostructural mapping of rock walls. These examples show that terrestrial night-time photogrammetry has been functioning for years, albeit with a small and narrow scope of applications. 

Night-time photogrammetry using UAVs has been developing for several years. The appearance of very sensitive digital camera sensors with low specific noise and small cameras working in the thermal infrared band made it quite easy to use them on UAVs [60,61]. So far, the main target of interest for UAV night-time photogrammetry has been urban lighting analyses. 

The problem of artificial lighting analysis may comprise of numerous aspects. The first one is safety [62]. This topic covers analysing the intensity of lighting, which is important from the perspective of local safety, and enables optimizing lamppost arrangement, designing illumination direction, power, and angle, and thus selecting lighting fixtures. A properly illuminated spot on the road allows to see danger in time, is cost-efficient, and does not dazzle drivers [63,64,65]. On the other hand, artificial lighting also has a negative impact on humans and animals [66]. It has been an actor in the evolution of nature and humans for a short time, and its presence, especially accidental, is defined as artificial light pollution [67]. The issue associated with this phenomenon is increasingly noticed already at society level [68]. Night-time spatial data will be an excellent tool for analysing light pollution. Data from digital cameras mounted on UAVs and data from terrestrial laser scanning (TLS) for analysing street lighting is used for this purpose [69]. The authors of [70] showed that UAV data enable capturing urban lighting dynamics both in the spatial and temporal domains. In this respect, the scientists utilized the relationship between observed quality and terrestrial observations recorded using a sky quality meter (SQM) light intensity tool. For the purposes of determining luminance, the authors of [71] present methods for calibrating digital cameras fixed on UAVs and review several measuring scenarios. 

UAVs equipped with digital cameras are becoming a tool that can play an important role in analysing artificial light pollution [72,73]. The possibility of obtaining high-resolution point clouds and orthophotoimages is an important aspect in this respect. The aforementioned research did not involve an in-depth analysis of the photogrammetric process and the geometric accuracy of typical models based on night-time photos (with existing street lighting). All analyses based on spatial data obtained through digital imaging should be supported with analysing point cloud geometric errors and evaluating the quality of developed orthomosaic. Night-time acquisition, as in the above cases, should not be treated the same as day-time measurements. These studies assumed, a priori, that typical photogrammetric software was equally good with developing photos taken in good lighting and night-time photographs. The issues that impact night-time image quality can also be scenery lighting discontinuity or too poor lighting at the scene, and increased levels of noise and blur appearing on digital images obtained from UAVs. 

As a result of the above considerations, the authors of this paper put forward a thesis that a photogrammetric product based on night-time photos will exhibit lower geometric accuracy and reduced interpretative quality. It seems that this thesis is rather obvious. However, after a deeper analysis of the photogrammetric imaging process, starting with data acquisition, through processing and to spatial analyses, it is impossible to clearly state which of the elements of this process will have the greatest influence on the quality of a photogrammetric product. This leads to questions concerning which process element had the greatest impact on degrading a night-time photogrammetric product, which measurable factor directly correlated with image accuracy, and whether commercial photogrammetric software enabled reaching acceptable image quality and whether the digital camera type impacted interpretative quality. Therefore, the authors of this study set a research objective of determining the impact of photogrammetric process elements on the quality of a photogrammetric product, for the purposes of identifying artificial lighting at night. 

## 2. Materials and Methods

### 2.1. Research Plan

**Figure 1:**
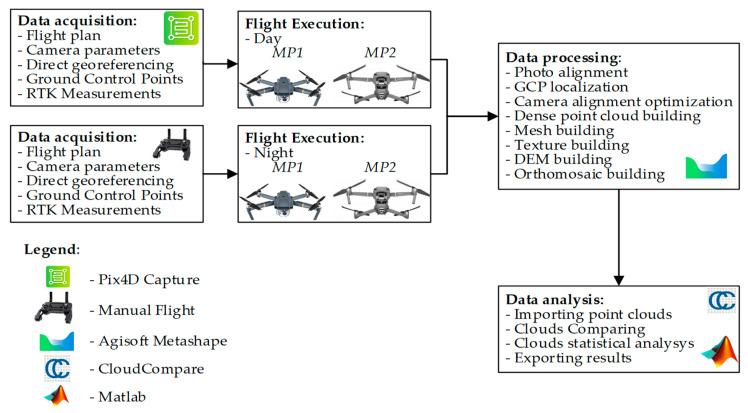
Test and result processing diagram.

In order to verify the thesis and research assumptions of this paper, a test schedule and computation process were developed. This process in graphic form, and the used tools, are shown in Figure 1. The test data were acquired using two commercial UAVs. The study involved 4 research flights, two during the day, around noon, and two at night. Study data were processed in popular photogrammetric software, and typical photogrammetric products were then subjected to comparative analysis. The study involved taking day-time photos in automatic mode, and night-time ones manually. This enabled correctly exposing the photos at night, without visible blur. The details of these operations are shown in the further section of this paper.

### 2.2. Data Acquisition

Research flights were conducted using two UAV types: DJI Mavic Pro (MP1) and DJI Mavic Pro 2 (MP2) (Shenzhen DJI Sciences and Technologies Ltd., Shenzhen, China). Such an unmanned aerial vehicles are the representatives of commercial aerial systems, designed and intended primarily for recreational flying and for amateur movie-makers. The versatility and reliability of these devices was quickly appreciated by the photogrammetric community. Their popularity results mainly from their operational simplicity and the very intuitive ground station software. 

Both UAVs are equipped with an integrated digital camera with a CMOS (complementary metal-oxide-semiconductor) sensor Table 1. An FC220 digital camera installed onboard the MP1 is a compact device with a very small 1/2.3″ (6.2 × 4.6 mm) sensor and a minor maximum ISO (1600) sensitivity. The more recent Hasselblad L1D-20c structure, installed onboard the MP2, is characterized by 4× the sensor area of 1” (13.2 × 8.8 mm) and a maximum ISO of 12800. In light of the technical specification of the L1D-20c camera, it can be presumed that it will provide greater flexibility at night, and will allow to obtain images of better quality.

The dimensions of the test area are 290 × 630 m, with all the flights conducted at an altitude of 100 m AGL (above ground level), with a forward and lateral overlap of 75%. Depending on the case, a total of 114 to 136 photos with metadata and the actual UAV position were taken. The data are saved in the EXIF (exchangeable image file format). Day-time flights were programmed and conducted automatically, according to a single grid plan, over the designated terrain. Nigh-time flights were conducted following a single grid plan, although in manual mode. In order to minimize blur induced by long exposure time at night, every time after taking the position to take the photo, the operator would hover for 2–3 s, and manually release the shutter after stabilizing the vehicle. The aim of this procedure was to obtain possibly sharp images without blur. All images acquired at night were visually assessed as sharp and without blur.

**Figure 2:**
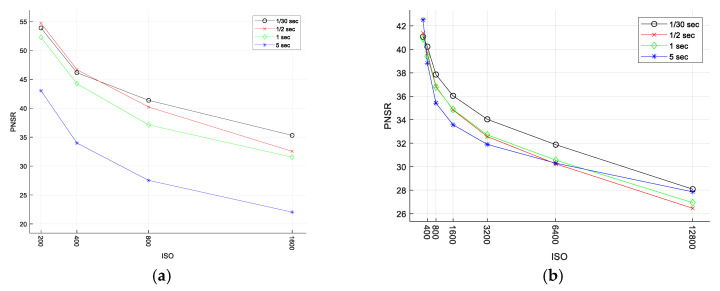
The PSNR ratio calculated for (**a**) MP1 and (**b**) MP2 and diferent shuter speed.

Sensor sensitivity was set manually at a constant value of 400 ISO, which extended the exposure time, but enabled minimizing noise. As is was deeply investigated by Roncella et al. in [74], the most affecting parameter on the overall performance of the photogrammetric system in extreme low-light is the ISO setting. The higher ISO always increases the level of noise of the images, making the matching process less accurate and reliable. The peak signal-to-noise ratio (PSNR), calculated as in [74] for test images (Figure 2, with reference to the ISO 100 test image, proves that the image quality reduces, and noise increases for higher ISO for utilized cameras. With the procedure used to take the image (while hovering), increasing the ISO above 400 would increase the noise, but without affecting the blur significantly. Reducing ISO below 400 resulted in longer exposure time and increased blur. The right ISO setting is a balance between the noise and the shutter speed for particular lighting conditions.

The white balance was also set manually to a fixed value. It should be noted that the digital camera used within this research can effectively take photos automatically both during the day and night. In automatic mode, the processor adapts all adjustable exposure parameters. In the case of the white balance, its value is also adjusted for each shot. As far as measurement night-time photos are concerned, exposure automation can be tricky. During the night, the processor attempts to maximize sensor sensitivity, at the expense of reduced exposure time and minimizing blur. This situation is exacerbated when flying over a study area without artificial lighting. The white balance can be changed even for every photo, especially, as seen in the presented case, when the urban lighting colour is variable. A summary of research flights is shown in Table 2.

**Figure 3:**
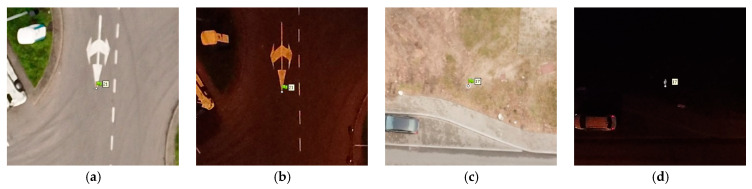
Day- and night-time control point image (**a**) MP1-D GCP No. 21, (**b**) MP1-N GCP No. 21, (**c**) MP1-D GCP No. 17, (**d**) MP1-N GCP No. 17.

A photogrammetric network was developed that comprised of 23 ground control points (GCPs), non-uniformly arranged throughout the study area, and their positions were measured with the GNSS RTK accurate satellite positioning method. The non-uniform distribution of points was forced directly by the lack of accessibility to the south-eastern and central parts of the area. The south-eastern area is occupied by the closed part of the container terminal, and the distribution of points in the central part, where the viaduct passes, was unsafe due to the high car traffic. GCP position was determined relative to the PL-2000 Polish state grid coordinate system and their altitude relative to the PL-EVRF2007-NH quasigeoid. All control points were positioned in characteristic, natural locations. These are mainly easyto identify in both day-time and night-time photos of terrain fragments with variable structure, road lane boundaries, and manhole covers. When locating control points, a priority rule that GCPs had to be located at spots illuminated with streetlamp lighting was adopted (Figure 3a,b). Furthermore, for analytical purposes, several points located within a convenient area were, however, not illuminated with artificial lighting (Figure 3c,d).

### 2.3. Data Processing

**Figure 4:**
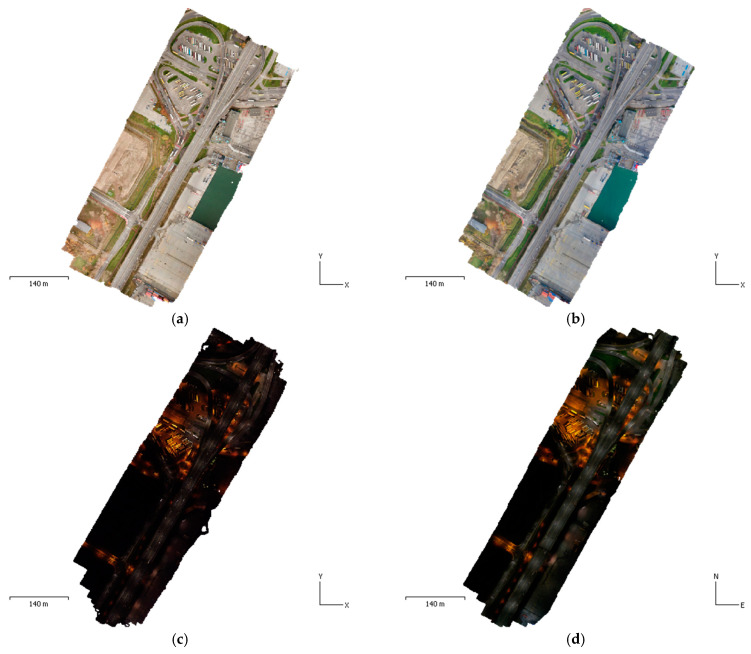
Developed orthophotoimages (**a**) MP1-D, (**b**) MP2-D, (**c**) MP1-N, (**d**) MP2-N.

The flights enabled to obtain images that were used to generate a photogrammetric product without any modifications. Standard products in the form of a point cloud, digital terrain model (DTM), and orthophotoimages in Figure 4 were developed in Agisoft Metashape v1.7.1 (Agisoft LLC, St. Petersburg, Russia).

Products were developed smoothly. The processing for each data set followed the same sequence, with the same settings. The operator tagged all visible GCPs both at night and day. Some GCPs at night were not readily identifiable on the photos due to insufficient lighting. Table 3 shows a brief summary of the basic image processing parameters. 

All of them contain a similar number of photos with the flight conducted at the same altitude of 100 m AGL (above ground level). As said above, MP1-N flight altitude is significantly underestimated, which is typical for photos of reduced quality, as demonstrated in [18]. Such an incorrect calculation is a symptom of errors that translate to the geometric quality of a product, which will be proven later in the paper. 

Table 4 shows a summary of tie point data. As can be seen, day-time products have a similar number of identified tie points, while night-time ones exhibit significantly fewer. 

Night-time data for the suggested analysis area enabled identifying approximately 30–40% tie points of the ones identifiable during the day. In addition, the mean key point size in the case of night-time photos is from 165% to 312% higher than the respective day-time cases.

Table 5 and Table 6 show the mean camera position error and root mean square error (RMSE) calculated for ground control point positions, respectively. The mean camera position error is the difference in the camera position, determined by an on-board navigation and positioning system resulting from aerotriangulation. The values of errors in the horizontal plane (*x*,*y*) fall within the limits typical for GPS receivers used in commercial UAVs. The absolute vertical plane error (*z*) is significantly higher (from 13 to 45 m) and arises directly from different altitude reference systems. Table 7 presents the root mean square error (RMSE) calculated for check points position.

**Figure 5:**
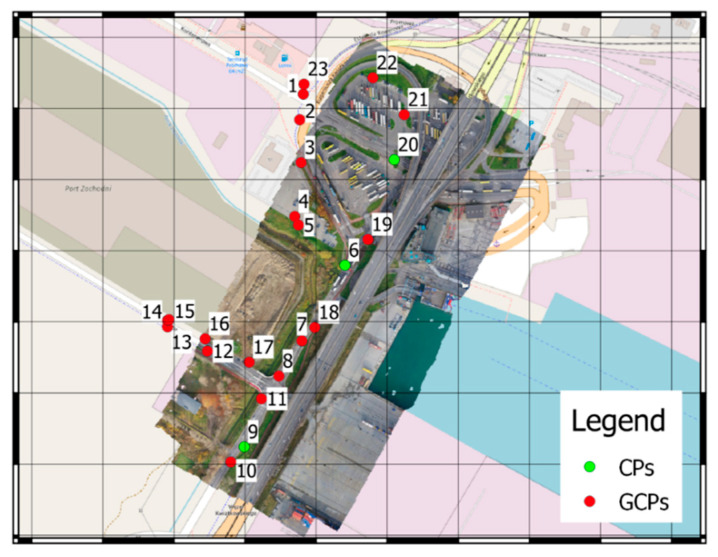
Ground Control Points (GCPs) and Check Points (CPs) distribution map.

A total of 26 ground control points were located in the field. All points visible on the photos were tagged by the operator and included in software calculations. Further, 13 and 14 points, respectively, were located in the processing area at daytime, while during the night 10 and 9 were visible. The points located in spots without lighting were not visible on the photos or their visibility was so low that it was impossible to properly mark them in the processing software with sufficient certainty. In order to control the quality of the process, three check points (CPs) were established and distributed over the study area. The GCPs and CPs distribution over the research area is presented in Figure 5.

### 2.4. Camera Calibration

The Agisoft Metashape software uses a parametric lens distortion model developed by Brown [75,76]. For a perspective pinhole camera, transformation of points $\left(X_{c},Y_{c},Z_{c}\right)$ in space ${ℜ}^{3}$ to coordinates in image plane (x,y) in space ${ℜ}^{2}$, omitting the final units of the image coordinate system, can be expressed as [77]:

(1) $$x=f\frac{X_{c}}{Z_{c}},$$

(2) $$y=f\frac{Y_{c}}{Z_{c}},$$

where *f* means the image plane distance from the projection centre. 

The camera’s local system has its origin in the centre of the camera system projections ($O_{c}$), axis $Z_{c}$ is oriented towards the observation direction, axis $X_{c}$ is oriented towards the right, axis $Y_{c}$ is oriented downwards, while the origin of the image coordinate system (background system) is located in the centre of the image plane. 

The camera’s optical system introduces certain deformations called distortions that cause displacement of point (x,y) within the background plane into a different position $\left(x^{\prime },y^{\prime }\right)$. The new position, taking into account radial and decentring distortion, can be expressed as:

(3) $$x^{\prime }=x\left(1+K_{1}r^{2}+K_{2}r^{4}+K_{3}r^{6}+K_{4}r^{8}\right)+\left(P_{1}\left(r^{2}+2x^{2}\right)+2P_{2}xy\right),$$

(4) $$y^{\prime }=y\left(1+K_{1}r^{2}+K_{2}r^{4}+K_{3}r^{6}+K_{4}r^{8}\right)+\left(P_{2}\left(r^{2}+2y^{2}\right)+2P_{1}xy\right),$$

where: $K_{1},\;K_{2},\;K_{3},\;K_{4}$ are radial symmetric distortion coefficients, $P_{1},\;P_{2}$ are decentring distortion coefficients (including both radial asymmetric and tangential distortions), while *r* is the radial radius, defined as:

(5) $$r=\sqrt{x^{2}+y^{2}}.$$

Because in the discussed case we are dealing with an image plane in the form of CMOS sensors, it is necessary to convert $\left(x^{\prime },y^{\prime }\right)$ into image coordinates expressed in pixels. In the case of digital images, coordinates are usually given in accordance with the sensor indexation system adopted in the digital data processing software. Therefore, for this image, axis *x* is oriented to the right, axis *y* downwards, and the origin is located at the centre of the pixel (1,1). Coordinates for this system are expressed in pixels. Therefore, taking into account the image matrix size, the physical size of the image pixel, affinity non-orthogonality, principal point offset, and the projected point coordinates in the image coordinate system, we can write:

(6) $$u=0.5w+c_{x}+x^{\prime }f+x^{\prime }B_{1}+y^{\prime }B_{2},$$

(7) $$v=0.5h+c_{y}+y^{\prime }f,$$

where: u, v is expressed in pixels (px), f denotes focal length (px), $c_{x}$, $c_{y}$ principal point offset (px), $B_{1},\;B_{2}$ affinity and non-orthogonality (skew) coefficients (px), w, h image width and height, image resolution (px), and they are all defined as intrinsic orientation parameters (IOP).

Correct determination of IOPs (intrinsic orientation parameters) and distortion parameters is very important in terms of photogrammetry. In the traditional approach, these parameters are determined at a laboratory and are provided with a metric camera. UAV photogrammetry usually uses small, non-metric cameras, where the intrinsic orientation and distortion parameters are unknown and unstable (not constant and can slightly change under the influence of external factors like temperature and vibrations). Due to the fact that the knowledge of current IOPs is required, the photogrammetric software used in this study calibrates the camera in each case, based on measurement photos, and calculates current IOPs for given the conditions and camera. Such a process ensures result repeatability. However, it functions correctly mainly in the case of photos taken under good conditions, i.e., during the day, sharp, and well-illuminated. Table 8 shows IOPs and distortion parameters determined by the software for each case.

## 3. Results

### 3.1. Geometry Analysis

**Figure 6:**
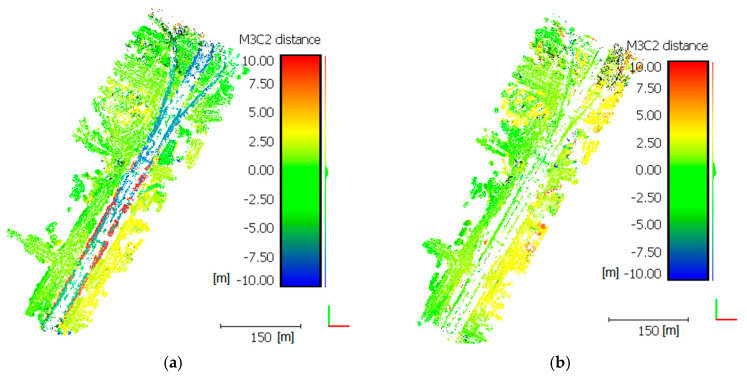

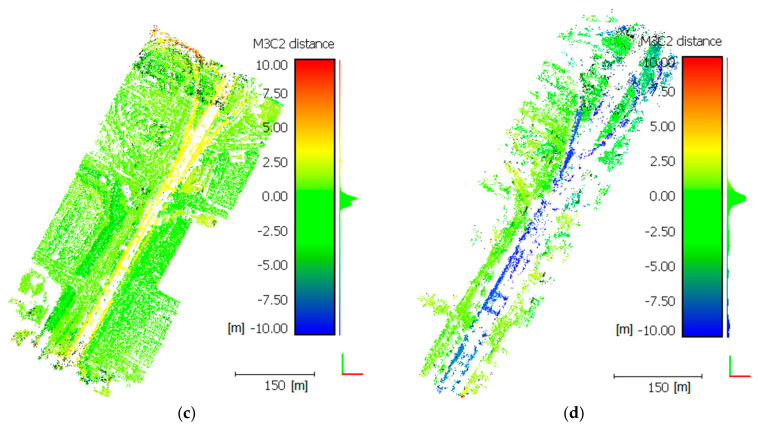
M3C2 Distances (**a**) MP1-D/ MP1-N, (**b**) MP2-D/ MP2-N, (**c**) MP1-D/ MP2-D, (**d**) MP1-N/ MP2-N.

The geometric quality of developed relief models was evaluated using the methods described in [78]. An M3C2 distance map (multiscale model to model cloud comparison) was developed for each point cloud. The M3C2 distance map computation process utilized 3-D point precision estimates stored in scalar fields. Appropriate scalar fields were selected for both point clouds (referenced and tested) to describe measurement precision in X, Y, and Z ($\sigma_{X}$, $\sigma_{Y}$, $\sigma_{Z}$). The results for the cases are shown in Figure 6.

The statistical distribution of M3C2 distances is close to normal, which means that a significant part of the observations is concentrated around the mean (Table 9). The mean (µ) for the comparison of night-time and day-time cases shows, both from MP1 and MP2, that the models are 0.4 to 1.6 m apart. Furthermore, standard deviation (σ) (above 4 m) indicates that there are significant differences between the models. A visual analysis Figure 6 explicitly shows that the highest distance differences can be seen in the area of the flyover, which is a structure located much higher than the average elevation of the surrounding terrain in the case of the flight with UAV MP1 (Figure 6a). The same comparison of the night-time and day-time models for UAV MP1 does not exhibit such significant differences in this area (Figure 6b). It should be noted that M3C2 values are clearly higher in the area remote from GCP (south-eastern section of Figure 6a,b, values in yellow). It follows directly that, within the model development process, upon a significantly reduced tie point number, which is the case for night-time photos, aerotriangulation introduces a significant error. This is error is greater the farther the tie points are from identified GCPs. This phenomenon does not occur to such an extent for day-time cases (day MP1/day MP2) shown in Figure 6c. It is confirmed by the visual and parametric assessment of statistical data (Table 9). 

The D-MP1/MP2 case exhibits values µ = 0.11 m σ = 2.52 m, and a clear elevation of the deviation occurs only in the flyover area. The analysis of the night/night case clearly emphasizes the issue of reconstructing higher structures (flyover, building), which is particularly based on correct aerotriangulation. The error of reconstructing objects located higher than the average terrain elevation, with rapidly increasing elevation is significant. This confirms that a reduction in the tie point number caused by low intensity has a great impact on reconstruction errors. It is clearly demonstrated in the area of the flyover highway, where the software does not identify so many tie points.

### 3.2. Autocalibration Process Analysis

**Figure 7:**
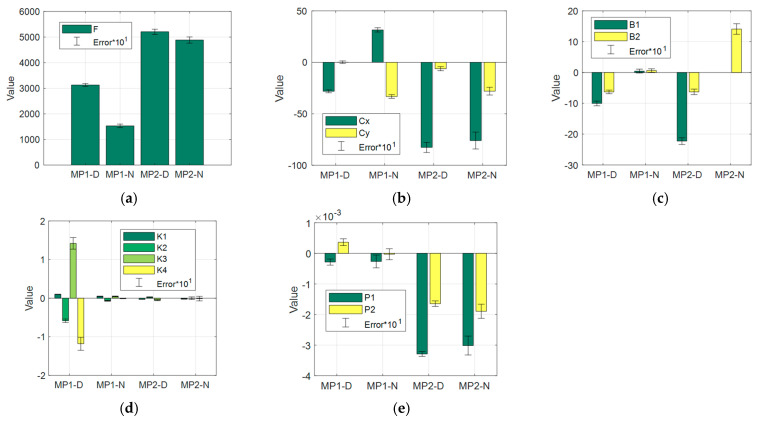
Graphical comparison of calibration parameters (**a**) F, (**b**) Cx and Cy, (**c**) B1 and B2, (**d**) K1-4, (**e**) P1 and P2.

Intrinsic orientation parameters (IOPs), including lens distortion parameters, have a significant impact on the geometry of a photogrammetric product. The automatic calibration process executed by Agisoft is based primarily on correctly identified tie points. Figure 7 shows a graphical comparison of the parameters, previously shown in Table 8. The graphical presentation of grouped results with corresponding error, for day/night cases, shows a noticeable trend and relationships that translate to the above reduced image quality. 

Theoretically, and in line with previous experience [18,19,79], the calibration parameters for a single camera should be the same or very similar. Typically, especially for non-metric cameras, the recovered IOP are only valid for that particular project, and they can be slightly different for another image block under different conditions. In the case of night-time calibration, which can be seen in Figure 7, IOPs are significantly different than in the case of day-time calibration. This difference is particularly higher for cameras with lower quality and sensitivity (UAV MP1). The focal length (*F*) for MP1 changes its value by 50% during the night, and only by 6% in the case of MP2. Tie point location ($C_{x},C_{y}$) for MP1 was displaced by more than 60 pixels in the *x*-axis during the night. B coefficients for night-time cases tend to strive to zero. We can also observe significant differences in terms of radial and tangential distortion for UAV MP1. Whereas the distortion parameters for the MP2 camera remain similar, they exhibit a higher error at night. The intrinsic orientation and distortion parameter determination error is significantly higher at night in all cases.

**Figure 8:**
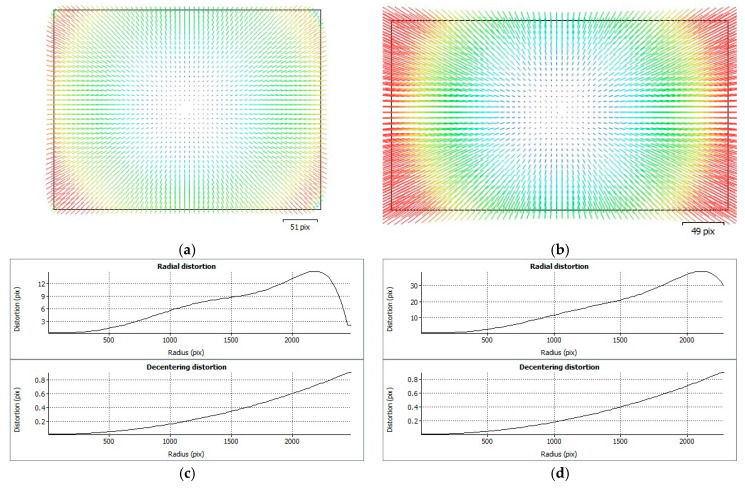
Distortion visualisations and profiles for MP1 day (**a**,**c**) and night cases (**b**,**d**).

**Figure 9:**
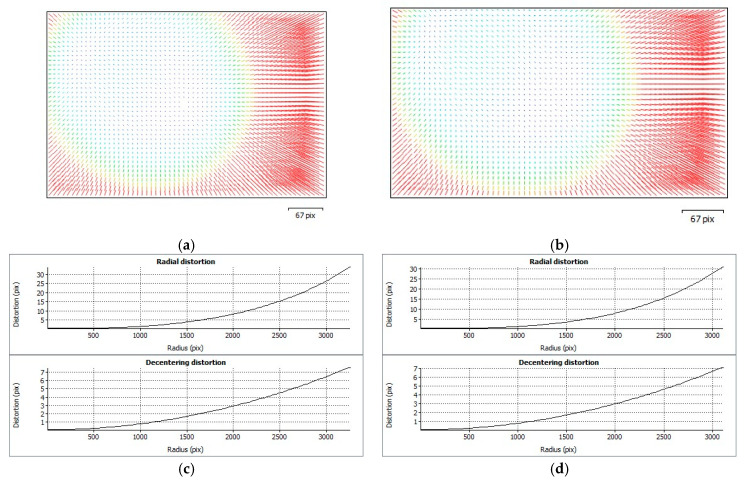
Distortion visualizations and profiles for MP2 day (**a**,**c**) and night cases (**b**,**d**).

In order to achieve additional comparison of calibration parameters, their distribution within the image plane and a profile depend on the radial radius. Figure 8 and Figure 9 show corresponding visualisations for MP1 and MP2, respectively.

**Figure 10:**
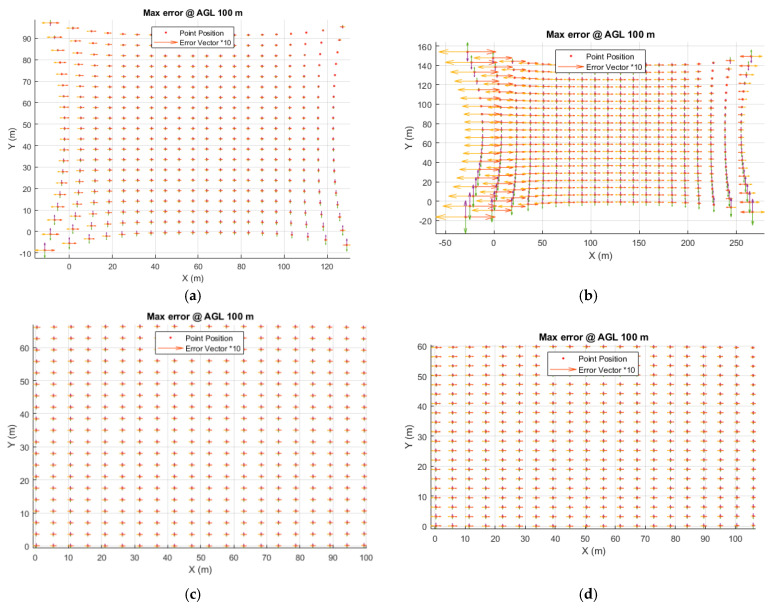
Simulated maximum ground point displacement for a flight altitude of 100 m, taking into account IOP and distortion parameter determination errors (**a**) MP1-D, (**b**) MP1-N, (**c**) MP2-D, (**d**) MP2-N.

As noted, the IOP and distortion determination error is significantly higher at night in each case. This means that these values are determined less precisely than during the day, which directly translates to reduced model development precision. In order to verify this hypothesis, the authors conducted additional analyses and calculated the maximum possible point ground displacement for a single air photo at an altitude of 100 m, taking into account the value of intrinsic orientation and distortion parameters increased by the maximum error value reported by photogrammetric software. In other words, formulas (1) to (7) were used to convert *u* and *v* to the spatial position of the points $\left(X_{c},Y_{c},Z_{c}\right)$ for a flight altitude of 100 m, considering IOPs and distortion parameters increased by the maximum error. The result of this operation is shown in Figure 10.

The statistical values of the maximum error in metres, for a flight altitude of 100 m, are shown in Table 10.

As shown in Table 10 the maximum displacement of ground points can occur in the MP1-N case and can even exceed 3 metres, with the mean displacement of approximately 60 cm. This displacement, resulting from the occurrence of a maximum error, is only informative, and according to Gaussian distribution, achieving such a situation in real life is very unlikely. Nonetheless, the increased error analysis conducted during night-time calibration proves that methods functioning correctly during the day are not able to accurately determine IOPs at night.

### 3.3. Relationships

**Figure 11:**
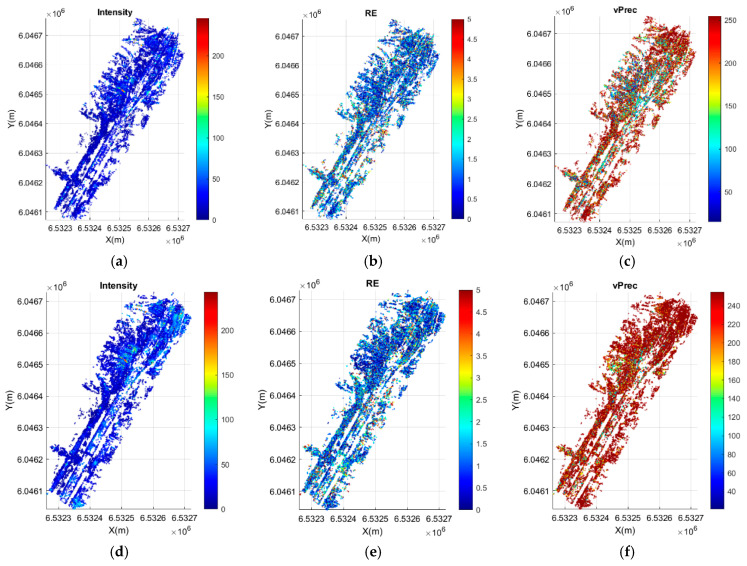
Tie point quality measurements—night cases (**a**) MP1 intensity, (**b**) MP1 RE (**c**) MP1-vPrec (**d**) MP2 intensity, (**e**) MP2 RE (**f**) MP2-vPrec.

Spatial relationships between point intensity, reprojection error and tie point determination precision are shown in Figure 11. The intensity map was calculated for each tie point ($I_{i}$) according to the formula [80]:

(8) $$I_{i}=0.21\;R+0.72\;G+0.07\;B,$$

where: *i* mean a tie point number, and *R*, *Gm* and *B* represent spectral response values recorded by a camera sensor for a given tie point and a red, green, and blue spectrum, respectively.

In order to evaluate the tie point precision distribution, the authors introduced a value of the precision vector (vPrec—$d_{\sigma }$), such that:

(9) $$d_{\sigma_{i}}=\sqrt{{\left(\sigma_{X}{}_{i}\right)}^{2}+{\left(\sigma_{Y}{}_{i}\right)}^{2}+{\left(\sigma_{Z_{i}}\right)}^{2}},$$

where: $\sigma_{X}$, $\sigma_{Y}$, $\sigma_{Z}$ means the measurement precision for the *i*-th tie point, obtained throug the method described in [78]. These values are calculated in millimetres (mm).

**Figure 12:**
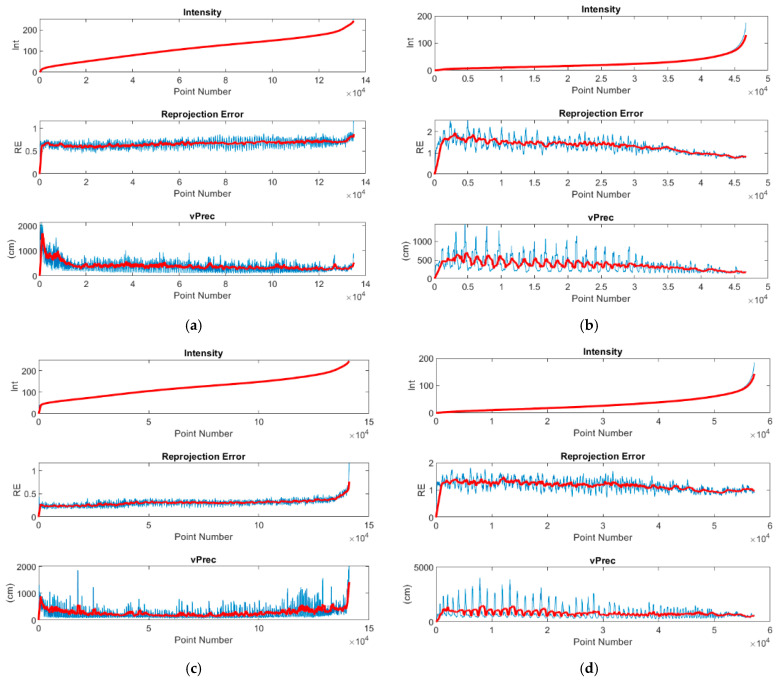
Correlations between accuracy parameters relative to point intensity (**a**) MP1 Day (MP1-D), (**b**) MP1 Night (MP1-N), (**c**) MP2-Day (MP2-D), (**d**) MP2-Night (MP2-N).

Figure 12 shows the relationships of the $d_{\sigma_{i}}$ precision vector (vPrec) reprojection error and intensity, calculated for each tie point. The blue line represents accurately obtained values, while the red line averages them in order to visualise the trend and relationships. All data were sorted in ascending order of intensity.

The mean value for models based on day-time photos is 113 and 124 with a standard deviation of 51 and 44, respectively (Table 11). The mean RE value for day-time models is 0.6656 px ($s_{RE\left(MP1D\right)}=0.5253$) and 0.3130 px ($s_{RE\left(MP2D\right)}=0.3659$), respectively. The maximum RE value for day-time models does not exceed 21 pixels. The $s_{RE}$ analysis for day-time cases indicates that values are relatively constant for all points, which is also confirmed by the graph (Figure 12). The mean value for models based on night-time photos is 25 and 32 with a standard deviation of 23 and 27, respectively. The mean RE value for night-time models is 1.3311 px ($s_{RE\left(MP1N\right)}=1.407$) and 1.168 px ($s_{RE\left(MP2N\right)}=1.26$), respectively, and the maximum RE value is significantly higher at approximately 37 pixels. Conversely, for night-time cases, $s_{RE}$ takes higher values, which proves high result variability. This can be easily seen in the graphs (Figure 12), where the blue plot for RE and vPrec for night-time cases shows a high amplitude change. 

An analysis of relationships between intensity, RE and vPrec shows a strong correlation between accuracy parameters in nigh-time photos, i.e., those, where the mean intensity is in the range from 25 to 35. On the other hand, the same relationships are not exhibited by models based on day-time photos. The results show that RE stabilizes towards the mean only above an intensity of 30 and 50, respectively.

A decrease in the number of tie points following a decrease of intensity results directly from the algorithm applied at the first stage of the photogrammetric stage, namely, feature matching. The software developer uses a modified version of the popular SIFT (scale-invariant feature transform) algorithm [81]. As shown by the studies in [82,83,84,85,86,87], the SIFT algorithm is non-resistant to low intensity and low contrast. This leads to the generation of a certain number of false key points, which are later not found on subsequent photos, thus becoming tie points.

**Figure 13:**
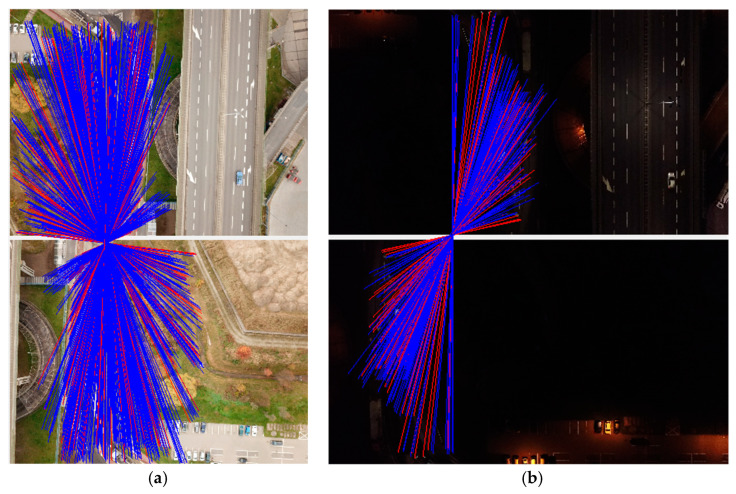
Decrease in the number of matches for day- and night-time cases (**a**) 1238 total matches, (**b**) 208 total matches.

The maximum total number of matches in MP1 night-time photos is 1080, including 1018 valid and 62 invalid matches. Similarly, the maximum total number of matches for day-time photos is 3733, including 3493 valid and 240 invalid matches. A decrease in the number of matches by more than 70% greatly impacts image quality. This phenomenon was demonstrated for a pair of identical photos in the same region in Figure 13. The figure shows a day-time example of a stereopair from 1238 total matches, and a night-time stereopair from 208 total matches.

## 4. Discussion

The results and analyses conducted within this study show the sensitivity of a photogrammetric process applied in modern software, relative to photos taken in extreme lighting conditions. The issues analysed by the authors can be divided as per the division in [18], intro procedural, numerical and technical factors. Procedural factors that impact the final quality of a photogrammetric product include GCP distribution and flight plan, whereas in terms of numerical factors, it is the issue of night-time calibration. Thirdly, presented technical factors included the difference between cameras on two different UAVs. 

The reduction in geometry during night-time tests were greatly impacted by non-uniform distribution of GCPs within the study area. As was noted, wherever there were no placed GCPs, the distance between the reference model and the night-time model significantly increased. In the case of day-time photos, these differences are not as important since this phenomenon is compensated by high tie point density and their low mean diameter. As recommended by most photogrammetric software developers, 5–10 ground control points should be arranged uniformly in such a case. This ensures acceptable product quality. The situation is quite the opposite at night, when the tie point density decreases by up to 70%, which results in the formation of aerotriangulation errors. A night-time study will lead to the reprojection error increasing with growing distance from GCPs and with a change in object height. In the case in question, it was physically impossible to uniformly arrange GCPs, due to safety (traffic on the flyover—expressway) and in the south-eastern area, which is a closed seaport section. The location of the research in such a challenging area was not a coincidence. The authors decided on this location because there is indeed a problem with light pollution in this area and there is a wide variety of urban lighting of different types. The location of the study is closest to the real problems that can be encountered during the measurements and can induce new and interesting challenges for science. After all, it is not always physically possible to evenly distribute GCPs, which during the day does not induce significant problems, but as shown, at night can be a rather more significant factor.

GCP arrangement at night should be properly planned. Objects intended to serve as natural or signalled points should be illuminated. This is a rather obvious statement. However, it requires a certain degree of planning when developing the photogrammetric matrix and, more importantly, such a selection of control points so that they are sufficiently contrasting. Low contrast of natural control points results in a significant reduction of identifiability at low lighting intensity. An active (glowing marker), new type of control point might be a good solution in insufficiently illuminated spots, where it is impossible to place or locate control points. 

A developed and executed night-time flight plan covered manual flight execution, following a single grid. The UAV had to be stopped every time for 1–3 s, until hover was stabilized, and the shutter released. The time required to stabilise the hover depends, of course, on the inertia of the UAV itself and its flight dynamics. Such a procedure enables taking a photo without clear blur and allows to optimally extend the exposure time, and reduce ISO sensitivity in order to avoid excessive matrix noise. It seems impossible to take sharp, unblurred photos without stopping in-flight, as is the case with a traditional day-time flight. Commercially available software for planning and executing UAV flights (Pix4D Capture—Android version) does not offer a function of automatic breaking during exposure, and furthermore, does not enable full control over exposure parameters. The same software offers the possibility to stop the UAV while taking picture only on iOS (mobile operating system created Apple Inc., Cupertino, CA, USA). Full control is possible also via UAV manufacturer’s dedicated software. However, it does not enable automatic flight plan execution. 

Commercial aerial systems are equipped with built-in non-metric digital cameras, the intrinsic orientation and distortion parameters of which are not constant. Such cameras require frequent calibration, which is practically achieved every time, with each photogrammetric process. This process, as demonstrated, is sensitive to poor lighting conditions, which leads to the generation a higher determination error for each intrinsic orientation and distortion parameter. Since it is these parameters that significantly influence the geometric quality of a product, their correct determination is extremely important. As indicated by studies, a brighter camera of a generally better quality, used onboard Mavic 2 Pro, exhibited clearly lower deviation of night-time calibration parameters, relative to the same settings during the day. This enables obtaining clearly more stable results.

As evidenced, uniformly illuminated photos can be used to construct a model with lower RE, and each tie point exhibits greater precision. The issue of decreasing precision at low photo brightness results from the type of algorithm used to detect tie points, which means that, potentially, improving this element within the software would improve the geometric quality of the model generated based on night-time photos.

## 5. Conclusions

As shown in the introduction, a number of publications on night-time photogrammetric products did not analyse the issue of model geometry. These studies assumed, a priori, that geometry would be similar to that during the day, and focused on the application-related aspects of night-time models. As indicated in this research, popular photogrammetric software generates night-time models with acceptable geometry. However, their absolute quality is poor. It can be concluded that the geometry of night-time models cannot be simply used for surveying or cartographic projects. This is greatly influenced by the image processing itself and used algorithm.

A more in-depth analysis of the photogrammetric process indicated that a decrease is experienced on many levels, starting with procedural factors (data acquisition, photogrammetric flight), through numerical factors (calibration process and algorithms applied to detect key points), to technical factors (camera type and brightness). These factors blend and mutually impact image quality. It cannot be clearly stated which of the factors has the greatest influence on the geometric quality of an image, although it seems obvious that a precise camera and a stable UAV flight will be the best combination. Therefore, it can be concluded that technical factors will be the most decisive in terms of night-time image quality. They will be followed by procedural factors. The very procedure of night-time photo acquisition must ensure taking a sharp photo, namely a stable hover during the exposure time. Such a solution will ensure a sharp photo without blur. Another element of the procedure is uniform GCP distribution in an illuminated location. Distribution uniformity has a significant impact on image geometry, especially with low tie point density. The last element is the numerical factor, which the user has little influence on in this case. Software does not allow to change applied algorithms or the calibration procedure, and one can guarantee conditions correct for the operation of these algorithms only through good practice. The biggest problem was noted in the terms of the calibration algorithm, with even a slight error increase resulting in significant geometric changes and ground point displacement, by up to several metres.

## Figures and Tables

**Table 1:** Technical data of camera installed onboard UAVs.

Technical Data	FC220	Hasselblad L1D-20c
Sensor size	1/2.3″ CMOS, 12.35 MP	1″ CMOS, 20 MP
Pixel Size	1.57 × 1.57 μm	2.41 × 2.41 μm
Focal Length	4.73 mm	10.26 mm
Lens	FOV * 78.8° (28 mm **) f/2.2	FOV * 77° (28 mm **) f/2.8
Focus	from 0.5 m to ∞	from 1 m to ∞
ISO Range (photo)	100–1600	100–12,800
Shutter Speed, type	8 s–1/8000 s, electronic	8 s–1/8000 s, electronic
Image Size (pixels)	4000 × 3000	5472 × 3648
Photo file format	JPEG, DNG	JPEG, DNG

* FOV—field of view, ** 35 mm equivalent focal length.

**Table 2:** Research flight parameters.

Flight Symbol	UAV	Daytime	Flight Plan	Coverage Area
MP1-D	DJI Mavic Pro	day	single grid auto	0.192 km²
MP2-D	DJI Mavic Pro 2	day	single grid auto	0.193 km²
MP1-N	DJI Mavic Pro	night	single grid manual	0.114 km²
MP2-N	DJI Mavic Pro 2	night	single grid manual	0.147 km²

**Table 3:** Flight plan data—read from reports.

Flight	Flying Altitude (Reported)	Ground Resolution	Number of Photos	Camera Stations
MP1-D	105 m	3.05 cm/px	129	129
MP2-D	127 m	2.26 cm/px	126	126
MP1-N	69.7 m	3.11 cm/px	136	136
MP2-N	118 m	2.24 cm/px	114	114

**Table 4:** Tie points and reprojection error data.

Flight	Tie Points	Mean Key Point Size	Projections	Reprojection Error	Max Reprojection Error
MP1-D	134.894	5.08186 px	450.874	0.952 px	20.844 px
MP2-D	141.035	3.26046 px	414.585	0.543 px	20.5249 px
MP1-N	46.870	8.41085 px	131.625	2.04 px	37.4667 px
MP2-N	57.392	9.15704 px	154.054	1.85 px	36.7523 px

**Table 5:** Average camera location error. X—Easting, Y—Northing, Z—Altitude.

Flight	X Error (m)	Y Error (m)	Z Error (m)	XY Error (m)	Total Error (m)
MP1-D	2.60381	2.36838	44.9193	3.51981	45.057
MP2-D	2.00514	2.68327	22.9008	3.34971	23.1445
MP1-N	1.09268	3.65444	25.9414	3.8143	26.2203
MP2-N	2.49058	1.48535	13.4276	2.89987	13.7372

**Table 6:** Control point root mean square error (RMSE). X—Easting, Y—Northing, Z—Altitude.

Flight	GCP Count	X Error (cm)	Y Error (cm)	Z Error (cm)	XY Error (cm)	Total (cm)
MP1-D	13	9.53984	11.439	2.57716	14.895	15.1163
MP2-D	14	11.8025	15.6751	3.22474	19.6216	19.8848
MP1-N	10	3.52971	2.80579	2.06805	4.50902	4.96066
MP2-N	9	8.96118	8.146	0.531357	12.1103	12.122

**Table 7:** Check point root mean square error (RMSE). X—Easting, Y—Northing, Z—Altitude.

Flight	CPs Count	X Error (cm)	Y Error (cm)	Z Error (cm)	XY Error (cm)	Total (cm)
MP1-D	3	8.48112	16.8594	15.6909	18.8725	24.5433
MP2-D	3	21.8543	34.1211	25.9101	40.5199	48.0957
MP1-N	3	2.36402	4.10461	2.97748	4.73671	5.5948
MP2-N	3	2.03436	8.85104	5.07036	9.08182	10.4013

**Table 8:** Camera internal orientation element value for each image.

Parameter	Flight							
	MP1-D		MP2-D		MP1-N		MP2-N	
	Value	Error	Value	Error	Value	Error	Value	Error
F	3127.860000	5.200000	5206.030000	10.000000	1532.460000	6.800000	4882.830000	12.000000
Cx	−28.166700	0.140000	−82.683800	0.490000	31.520100	0.210000	−75.996700	0.820000
Cy	0.162081	0.120000	−6.182140	0.200000	−33.175400	0.180000	−28.009800	0.370000
B1	−10.043200	0.076000	−22.277500	0.110000	0.457534	0.063000	-	-
B2	−6.308330	0.058000	−6.289880	0.088000	0.629884	0.057000	14.112600	0.170000
K1	0.098579	0.000490	−0.029047	0.000170	0.047936	0.000510	−0.021496	0.000610
K2	−0.585459	0.004500	0.026580	0.000780	−0.068125	0.001300	−0.002758	0.003500
K3	1.420050	0.015000	−0.051838	0.001400	0.043968	0.001200	−0.011242	0.006000
K4	−1.180670	0.017000	-	-	−0.009856	0.000370	-	-
P1	−0.000286	0.000010	−0.003291	0.000008	−0.000265	0.000021	−0.003015	0.000031
P2	0.000362	0.000011	−0.001647	0.000009	−0.000030	0.000018	−0.001895	0.000023

**Table 9:** Mean distance value and standard deviation for M3C2.

M3C2 Case	Mean (m)	Std.Dev (m)
MP1-D/N	−0.39	4.14
MP2-D/N	1.63	4.59
D-MP1/MP2	0.11	2.52
N-MP1/MP2	−3.17	5.04

**Table 10:** Statistical values of the maximum error, in metres.

	MP1-D			MP2-D			MP1-N			MP2-N		
	$E_{X_{C}}$	$E_{Y_{C}}$	$E_{Z_{C}}$	$E_{X_{C}}$	$E_{Y_{C}}$	$E_{Z_{C}}$	$E_{X_{C}}$	$E_{Y_{C}}$	$E_{Z_{C}}$	$E_{X_{C}}$	$E_{Y_{C}}$	$E_{Z_{C}}$
Max	0.4697	0.3570	0.0001	0.1019	0.0724	0.0000	3.0835	1.7038	0.0003	0.1768	0.1017	0.0001
Min	−0.1712	−0.1351	0.0001	0.0781	0.0568	0.0000	−1.2506	−0.9880	0.0003	0.0664	0.0375	0.0001
Mean	0.1073	0.0804	0.0001	0.0897	0.0642	0.0000	0.6104	0.3072	0.0003	0.1187	0.0689	0.0001
Range	0.6409	0.4921	0.0000	0.0238	0.0156	0.0000	4.3340	2.6917	0.0000	0.1105	0.0642	0.0000
Std	0.0691	0.0458	0.0000	0.0032	0.0018	0.0000	0.5651	0.2492	0.0000	0.0160	0.0071	0.0000

**Table 11:** List of statistical data (m- mean, s-standard deviation).

Flight	$m_{I}$	$s_{I}$	$m_{RE}$ (px)	$s_{RE}$ (px)	$m_{vPrec}$ (cm)	$s_{vPrec}$ (cm)
MP1-D	113.24	51.78	0.6656	0.5253	401.22	1458.20
MP2-D	124.76	44.90	0.3130	0.3659	290.63	1686.36
MP1-N	25.76	23.49	1.3311	1.4070	365.21	994.63
MP2-N	32.839	27.02	1.1680	1.26	803.42	2471.73
